# Clinical characteristics and disease-specific prognostic nomogram for primary gliosarcoma: a SEER population-based analysis

**DOI:** 10.1038/s41598-019-47211-7

**Published:** 2019-07-24

**Authors:** Song-Shan Feng, Huang-bao Li, Fan Fan, Jing Li, Hui Cao, Zhi-Wei Xia, Kui Yang, Xiao-San Zhu, Ting-Ting Cheng, Quan Cheng

**Affiliations:** 1 Department of Neurosurgery, Xiangya Hospital, Central South University, 87 Xiangya Road, Changsha, Hunan 410078 P.R. China; 2 Department of Hepatobiliary and Pancreatic Surgery, First Hospital of Jiaxing, First Affiliated Hospital of Jiaxing University, Jiaxing, Zhejiang P.R. China; 3 Department of Rehabilitation, the Second Xiangya Hospital, Central South University, 139 People Road, Changsha, Hunan P.R. China; 4 Department of Psychiatry, the Second People’s Hospital of Hunan Province, the Hospital of Hunan University of Chinese Medicine, Changsha, 410008 Hunan P.R. China; 5 Department of Neurology, Xiangya Hospital, Central South University, 87 Xiangya Road, Changsha, Hunan 410078 P.R. China; 6 Department of Digestive, the 174th Hospital of the PLA, Xiamen, Fujian P.R. China; 7 Department of Preventive Health Care, Xiangya Hospital, Central South University, 87 Xiangya Road, Changsha, Hunan 410078 P.R. China; 8 Department of Oncology, Xiangya Hospital, Central South University, 87 Xiangya Road, Changsha, Hunan 410078 P.R. China; 9 Department of Clinical Pharmacology, Xiangya Hospital, Central South University, 87 Xiangya Road, Changsha, Hunan 410078 P.R. China

**Keywords:** Surgical oncology, Risk factors

## Abstract

Because the study population with gliosarcoma (GSM) is limited, the understanding of this disease is insufficient. In this study, the authors aimed to determine the clinical characteristics and independent prognostic factors influencing the prognosis of GSM patients and to develop a nomogram to predict the prognosis of GSM patients after craniotomy. A total of 498 patients diagnosed with primary GSM between 2004 and 2015 were extracted from the 18 Registries Research Data of the Surveillance, Epidemiology, and End Results (SEER) database. The median disease-specific survival (DSS) was 12.0 months, and the postoperative 0.5-, 1-, and 3-year DSS rates were 71.4%, 46.4% and 9.8%, respectively. We applied both the Cox proportional hazards model and the decision tree model to determine the prognostic factors of primary GSM. The Cox proportional hazards model demonstrated that age at presentation, tumour size, metastasis state and adjuvant chemotherapy (CT) were independent prognostic factors for DSS. The decision tree model suggested that age <71 years and adjuvant CT were associated with a better prognosis for GSM patients. The nomogram generated via the Cox proportional hazards model was developed by applying the rms package in R version 3.5.0. The C-index of internal validation for DSS prediction was 0.67 (95% confidence interval (CI), 0.63 to 0.70). The calibration curve at one year suggested that there was good consistency between the predicted DSS and the actual DSS probability. This study was the first to develop a disease-specific nomogram for predicting the prognosis of primary GSM patients after craniotomy, which can help clinicians immediately and accurately predict patient prognosis and conduct further treatment.

## Introduction

Gliosarcoma (GSM) is a rare malignant brain tumour composed of both glial and sarcomatous elements, the incidence of which is between 1% and 8% of all malignant gliomas^1–3^. GSM was first described by Stroebe in 1895 as a variant of glioblastoma (GBM) and gained wide acceptance after Feigin *et al*. and Rubinstein *et al*. published their papers presenting several patients with this malignant tumour in detail^4–6^.

Due to the low incidence of GSM, there are few studies describing the patient characteristics, treatment regimen and prognosis of GSM. As a variant of GBM, primary GSMs are often managed in accordance with GBM guidelines (the Stupp protocol)^7^. However, even after receiving standardized treatment, the prognosis of GSM patients remains dismal, with a median overall survival ranging from 6.6 to 18.5 months^8–11^.

In this study, retrospective data including a total of 498 patients who underwent craniotomy between 2004 and 2015 were reviewed from the Surveillance, Epidemiology, and End Results (SEER) database. The clinical characteristics and independent prognostic factors were analysed by applying large patient numbers. A prognostic disease-specific nomogram was constructed and validated based on retrospective patient data from the SEER database.

The nomogram is a multivariate visualization prediction model that can incorporate different variables affecting prognosis^12^. Recently, the nomogram has been widely used to predict the prognosis of patients with malignant tumours^13–16^. However, to our knowledge, no published literature has proposed a nomogram to predict the prognosis of primary GSM patients after craniotomy. Therefore, our study intended to develop a nomogram that can be applied to individually assess the survival time of patients with primary GSM after craniotomy and to discuss different factors influencing the prognosis of GSM patients.

## Results

### Patients’ clinicopathologic characteristics

**Figure 1:**
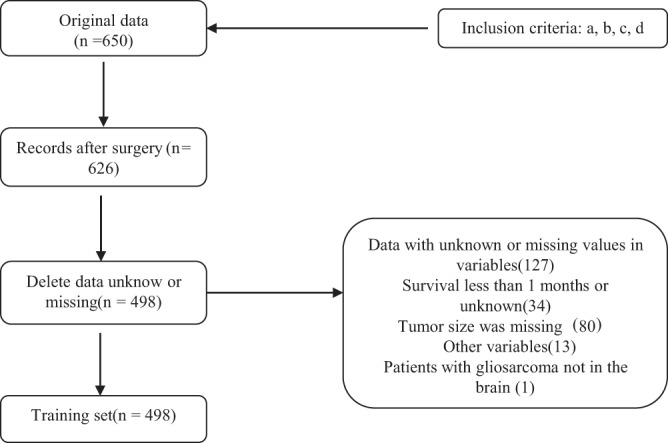
Flowchart of the selection criteria of patients with primary GSM.

**Figure 2:**
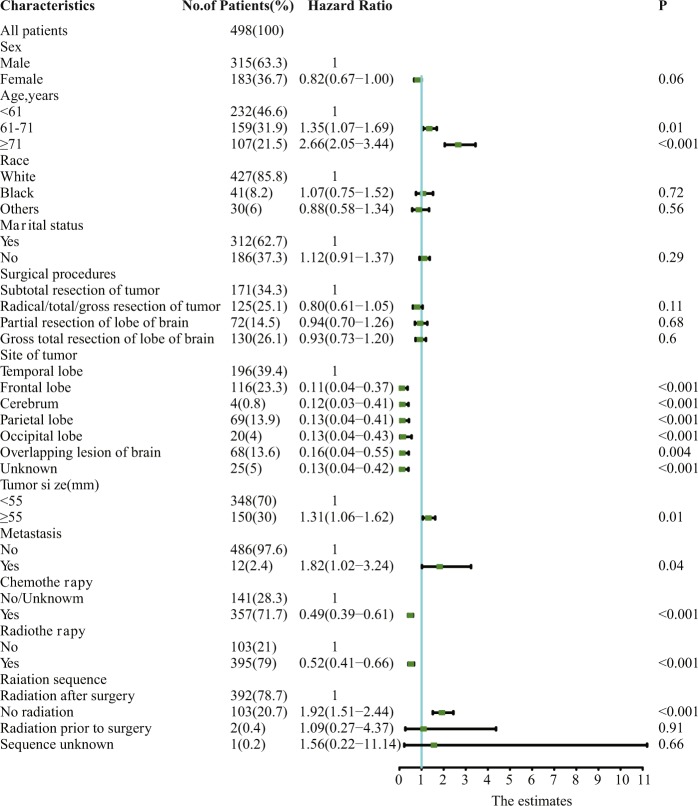
Patient and tumour characteristics and the univariate analysis of these factors on DSS (hazard ratio ± 95% confidence interval).

The study population consisted of 498 patients diagnosed with primary GSM receiving craniotomy. A flowchart of the case selection criteria of patients is shown in Fig. 1. Patient, tumour and surgical characteristics, including sex, age, race, marital status, surgical procedures, site of the tumour, tumour size, metastasis, chemotherapy and radiotherapy information, are displayed in Fig. 2. Most of the patients were male (315, 63.3%). Tumour metastasis was rare (12, 2.4%). The temporal lobe was more susceptible to tumours than other lobes (196, 39.4%).

### Disease-specific survival (DSS) and independent prognostic factors in the dataset

The median DSS was 12.0 months (range, 1.0 to 137 months), and the postoperative 0.5-, 1-, and 3-year DSS rates were 71.4%, 46.4% and 9.8%, respectively.

The univariate analysis is shown in Fig. 2. The results demonstrated that age at presentation, site of the tumour, tumour size, metastasis state, adjuvant chemotherapy (CT) and adjuvant radiotherapy (RT) were significantly associated with GSM patient survival. There was no significant difference regarding sex, race, marital status or surgical procedure.

**Figure 3:**
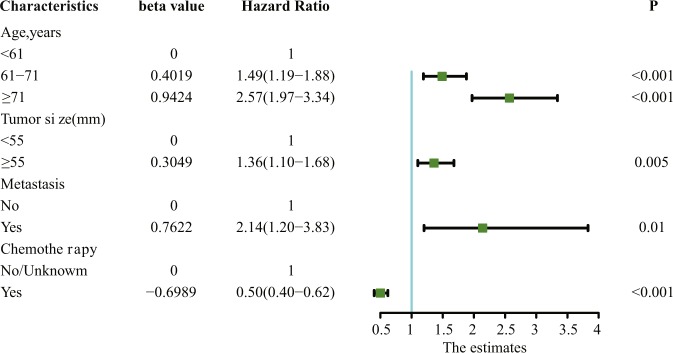
Multivariate analysis of different factors on DSS (hazard ratio ± 95% confidence interval).

**Figure 4:**
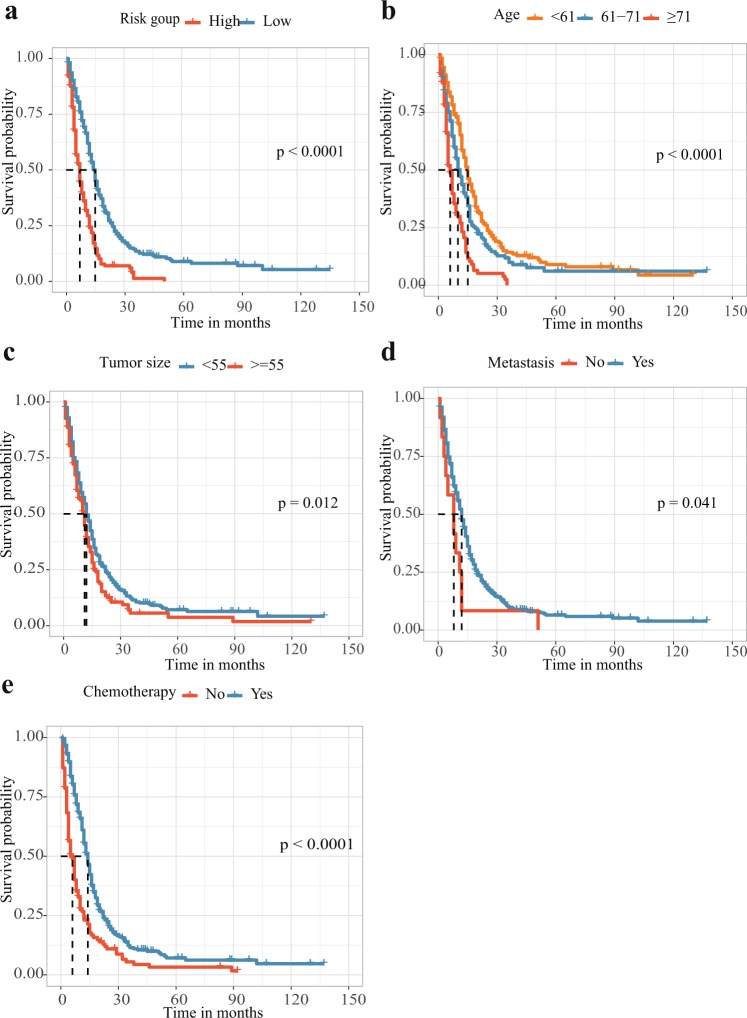
Kaplan-Meier DSS curves for patients with primary GSM according to different prognostic factors. (**a**) The disease-specific survival curve according to risk scores stratified into a high score group and a low score group by the average risk score. (**b–e**) Kaplan-Meier DSS curves for patients with primary GSM according to (**b**) age at presentation, (**c**) tumour size, (**d**) metastasis state and (**e**) adjuvant chemotherapy.

The results of the multivariate analysis are displayed in Fig. 3. Age at presentation (61–71 years: hazard ratio (HR) 1.49, 95% confidence interval (CI) 1.19–1.88, p < 0.001; ≥71 years: HR 2.57, 95% CI 1.97–3.34, P < 0.001), tumour size (≥55 mm: HR 1.36, 95% CI 1.10–1.68, P = 0.005), metastasis (HR 2.14, 95% CI 1.20–3.83, P = 0.01), and adjuvant CT (HR 0.50, 95% CI 0.40–0.62, P < 0.001) remained significant independent prognostic factors predicting GSM survival. Figure 4 displays the Kaplan-Meier DSS curves reflecting the independent prognostic factors for the patients with primary GSM.

### Prognostic nomogram for DSS and the decision tree model

**Figure 5:**
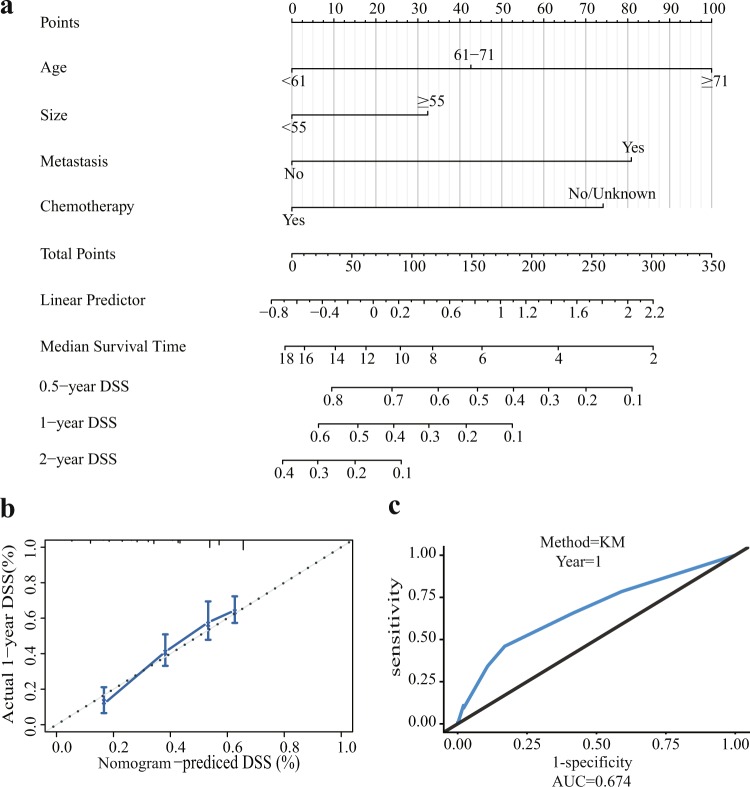
Nomogram, the internal calibration curve, and the ROC curve. (**a**) Nomogram for predicting the 0.5-, 1-, and 2-year disease-specific survival probabilities in patients with primary GSM following craniotomy. (**b**) The internal calibration curve for predicting 1-year disease-specific survival probability is displayed. The nomogram-predicted probability of DSS is plotted on the x-axis, and the actual probability of DSS is plotted on the y-axis. (**c**) The ROC curve shows the sensitivity and specificity of disease-specific survival prediction by the nomogram.

The nomogram generated via the Cox proportional hazards model included four independent prognostic factors influencing DSS after optimization by the Akaike information criterion (AIC) protocol, which is shown in Fig. 5. The C-index of internal validation for DSS prediction was 0.67 (95% CI, 0.63 to 0.70). The calibration curve for the probability of postoperative DSS at 1 year suggested that there was good consistency between the predicted DSS probability and the actual DSS probability in the dataset (Fig. 5). The receiver operating characteristic (ROC) curve and area under the curve (AUC) are displayed in Fig. 5. The AUC (0.67) indicated good accuracy of the one-year prognosis prediction of this model.

**Figure 6:**
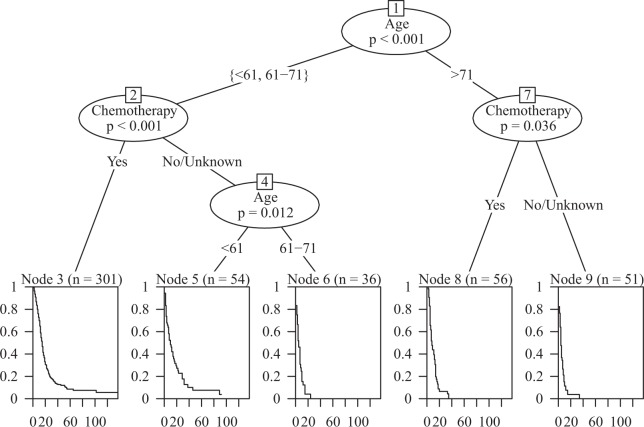
The decision tree model and two important parameters influencing GSM survival.

Figure 6 displays the decision tree model and two significant parameters influencing GSM survival (age and adjuvant CT).

## Discussion

Due to the rarity of this disease, data concerning the patient characteristics of primary GSM are lacking. Most previous studies have been based on single institutional experience, and the results do not represent the actual situation. Our results showed that the median age at diagnosis was over 60 years (61 years), and most of the patients were male (63.1%). The results were consistent with those from three other studies whose sample sizes included more than 50 patients^2,17,18^. The temporal lobe was more susceptible to tumours than other lobes (196, 39.3%). Most other previous studies also reported a tendency of temporal lobe involvement by GSM^2,17,19,20^. Ma R *et al*.^8^ reported that the tumours were most likely to involve the frontal and parietal lobes. However, there were only 33 patients in this study.

The multivariate analysis demonstrated that age at presentation, tumour size, metastasis state, and adjuvant CT were independent prognostic factors for DSS. Several other studies have also concluded that age at diagnosis was a significant prognostic factor and that a younger age was associated with a better prognosis^2,8,18^. To our knowledge, there have been no previous studies suggesting that tumour size is a prognostic factor. Our results showed that smaller tumours implied a better prognosis. Regarding the metastatic state, our study suggested that patients with tumour metastasis had a worse prognosis, which was verified in another study^21^.

There are no standardized management protocols for GSM. Generally, maximal surgical resection and adjuvant therapy are recommended^22^. Kozak *et al*. suggested that biopsy alone resulted in worse survival than either subtotal resection or gross total resection (GTR)^2^. Another study found that GTR resulted in better survival than subtotal resection or biopsy in GSM patients^18^. Our series did not find a significant difference in prognosis based on the surgical procedure. Regarding adjuvant therapy, trimodality therapy is considered the most effective method for GBM^7^. For low-grade gliomas (LGGs), the effect of adjuvant chemotherapy or radiotherapy alone was compared, and one study suggested that CT alone was associated with better survival than RT alone in patients with LGGs who received craniotomy^23^. Concerning GSM, previous studies have reached different conclusions. Some studies have concluded that chemotherapy is a prognostic factor^10,11,24^, while some have demonstrated that radiotherapy affects prognosis^2,17^, and others have indicated that trimodality therapy is the most beneficial for prognosis^8,9,18^. Our series found a significant correlation between chemotherapy and patient prognosis. We summarize several studies discussing the prognostic factors of GSM patients in Table 1.

**Table 1:** Studies reporting survival data and prognostic factors for GSM patients.

Study	Year of publication	No. of patients	Survival data	Prognostic factors
Kozak *et al*.^2^	2009	353	9 months (median OS)	Younger age, RT, Extent of resection
Singh *et al*.^11^	2012	22	18.5 months (median OS)	TMZ
Walker *et al*.^10^	2013	46	12.5 months(median OS)	TMZ
Rath *et al*.^24^	2015	27	16.7 months (median OS)	TMZ
Castelli *et al*.^17^	2016	75	13 months (median OS)	RT, Treatment at recurrence
Adeberg *et al*.^9^	2016	37	13.4 months (median OS)	Trimodality therapy
Ma *et al*.^8^	2017	33	6.6 months (median OS)	Age <50 years, Trimodality therapy
Frandsen *et al*.^18^	2018	1102	10.7 months (median OS)	Age <65 years, Female sex, Fewer comorbidities, Trimodality therapy, GTR
Our series		498	12.0 months (median DSS)	Age at presentation, Tumour size, Metastasis state, Chemotherapy

Although it is generally believed that the prognosis of GSM patients is poor, there are still reports of GSM patients with a relatively good prognosis. Huo Z *et al*.^25^ reported two cases of primary GSM with a prolonged prognosis (130 months and 48 months). Both patients received complete tumour resection and postoperative adjuvant therapy without any evidence of tumour recurrence or metastasis. Another case report presented a female GSM patient who was in stable condition at 31 months after the initial diagnosis^26^. Tumour resection and concomitant adjuvant therapy were performed after the initial diagnosis. Another surgery and second-line chemotherapy (ifosfamide, carboplatin, and etoposide) were conducted after tumour recurrence at 8 months. The authors discussed the feasibility of unconventional chemotherapy in the treatment of GSM.

Many prognostic models have been reported for different types of tumours. Breast cancer is the most common tumour in women, and the prognosis varies greatly. Phung MT *et al*.^27^ conducted a systematic review of studies discussing the prognostic models of breast cancer and identified 58 relevant models between 1982 and 2016. Within these 58 models, many methods of model development were applied. The most commonly used method was the Cox proportional hazards regression (n = 32). Other kinds of methods included an artificial neural network (n = 6), a decision tree (n = 4), logistic regression (n = 3), the Bayesian method (n = 3), a multistate model (n = 2), a support vector machine (n = 2) and others (n = 6). Four models applied a nomogram as the presentation form. When assessing discrimination ability, the C-index/AUC was the most commonly used method. Another systematic review of predictive models for resectable pancreatic cancer reported that within the 16 developed models, 11 used the Cox regression method^28^. There are also reports of the application of machine learning in the development of a clinical prognostic model^29,30^. However, the Cox proportional hazards regression method is still the most widely used method when establishing prognostic models.

In this study, we applied both the Cox proportional hazards model and the decision tree model to determine the prognostic factors of primary GSM and developed a prognostic DSS nomogram. By applying this nomogram, clinicians can immediately and accurately predict patient prognosis, which can help conduct further treatment after craniotomy. Regarding the decision tree model, we found that age and chemotherapy were important nodes for prognostic judgement. A younger age and adjuvant chemotherapy were associated with better survival for GSM patients.

We know that the ability to accurately predict patient outcome is important, yet the statistical methodology to assess the accuracy of these predictive models seems to be insufficient. Schumacher M *et al*.^31^ illustrated that the Brier score and the prediction error curves based on it are valuable for assessing the predictive performance of prognostic classification schemes through the analysis of two studies on node-positive breast cancer patients. The same study provided a more comprehensive perspective for clinical researchers to conduct these prognosis prediction studies and could help researchers select more appropriate statistical models based on the prediction error curves. The authors compared the predictive ability of different statistical methods (fuzzy inference, logistic regression, classification and regression tree) in another study^32^.

There are several limitations to our study. As a retrospective study, a selection bias was unavoidable. The use of the open access data from the SEER database provided a large amount of patients and surgical information, but several important factors affecting patient prognosis, including molecular/pathological information, were not available through this database. It is generally recognized that molecular pathological data, such as MGMT, are also associated with patient prognosis^18,33^. Thus, the prognostic factors we analysed based on the SEER database were not complete. The prognostic disease-specific nomogram developed in this study should undergo further improvements after adding these relevant data. Additionally, due to the rarity of this disease, we could not find sufficient clinical data to externally validate this nomogram.

## Material and Methods

### Patients and study design

A total of 498 patients receiving craniotomy between 2004 and 2015 were extracted from the 18 Registries Research Data of the SEER database. All patients were diagnosed with GSM by a histopathological examination. The variables included sex, age at diagnosis, race, marital status at diagnosis, surgical procedures, tumour size, primary site, metastasis state, and adjuvant therapy. The end of the follow-up was Dec. of 2015, and the primary endpoint was cause-specific death.

The inclusion criteria were as follows: (a) Primary site of the tumour: brain (CS Schema v0204+: brain); (b) Histologic type: GSM (ICD-O-3: 9442); (c) The tumour was the first and there was no other malignant tumour history (sequence number: one primary only; first malignant primary indicator: yes); and (d) The patient received surgery.

The exclusion criteria were as follows: (a) Survival was less than 1 month or unknown (according to clinical practice, patients who die within one month after craniotomy usually die of surgical complications; therefore, it may not be appropriate to incorporate these patients into a prognostic analysis.); (b) Tumour size was missing; (c) One patient had GSM that was not in the brain; and (d) Another variable was unknown or missing. The exclusion process is shown in Fig. 1.

### Statistical analysis

The continuous variables were transformed into categorical variables to match with the nomogram. The best cut-off points of continuous variables were identified with X-tile^34^. Categorical variables were grouped according to clinical reality. The DSS rate and the median DSS were calculated with the life table method.

Both univariate and multivariate Cox proportional hazard models were applied to calculate the HRs and their 95% CIs to analyse different prognostic variables associated with DSS^35^. Variables were included in the multivariate analysis if they reached a p value of ≤0.20 on the univariate analysis. These prognostic factors were screened with a Cox proportional hazard model adopting the bidirectional elimination method and were optimized with the AIC protocol^36^. The risk scores were then calculated according to the following formula: risk score = β1 × 1 + β2 × 2+ … +βnXn (β, regression coefficient; X, prognostic factors). Kaplan-Meier curves were plotted to compare DSS on account of different prognostic factors.

A nomogram was developed based on the independent prognostic factors and by using the rms package in R version 3.5.0 (http://www.r-project.org/). The discrimination of the nomogram was assessed by Harrell’s C-index, which could estimate the probability between the observed and predicted DSS^37^. A random resampling procedure (bootstrapping) with 1,000 resamples was used for internal validation. The ROC curve and the AUC were evaluated using the survivalROC package in R version 3.5.0 to assess the accuracy of one-year prognosis prediction. We also performed the decision tree model by using the party package in R version 3.5.0 to analyse the prognostic factors from other perspectives. P < 0.05 was considered statistically significant.

### Ethical declaration

This article does not contain any experiments on humans as well as animals and/or the use of human tissue samples performed by any of the authors.

## Conclusion

Our study was the first to develop a disease-specific nomogram to predict the prognosis of primary GSM patients after craniotomy based on retrospective patient data from the SEER database. This predictive model included four independent prognostic factors influencing DSS: age at presentation, tumour size, metastasis state, and adjuvant chemotherapy. Further research is needed to improve this nomogram by analysing more comprehensive prognostic data, and the effectiveness of this model should be evaluated in future clinical applications. Apart from the Cox proportional hazard model, we also performed the decision tree model to analyse the prognostic factors and determined that age and adjuvant CT were important prognostic factors.

## Supplementary information

Editorial Certificate
